# Risk Factors for Surgical Site Infection in Neonates: A Systematic Review of the Literature and Meta-Analysis

**DOI:** 10.3389/fped.2019.00101

**Published:** 2019-03-29

**Authors:** Vincenzo Davide Catania, Alessandro Boscarelli, Giuseppe Lauriti, Francesco Morini, Augusto Zani

**Affiliations:** ^1^Division of General and Thoracic Surgery, The Hospital for Sick Children, University of Toronto, Toronto, ON, Canada; ^2^Neonatal Surgery Unit, Department of Medical and Surgical Neonatology, Bambino Gesù Children's Hospital, IRCCS, Rome, Italy; ^3^Department of Pediatric Surgery, Spirito Santo Hospital and G. d'Annunzio University of Chieti and Pescara, Chieti, Italy

**Keywords:** newborn, wound infection, neonatal surgery, risk factors, systematic review, meta-analysis

## Abstract

**Purpose:** Surgical site infections (SSI) contribute to postoperative morbidity and mortality in children. Our aim was to evaluate the prevalence and identify risk factors for SSI in neonates.

**Methods:** Using a defined strategy, three investigators searched articles on neonatal SSI published since 2000. Studies on neonates and/or patients admitted to neonatal intensive care unit following cervical/thoracic/abdominal surgery were included. Risk factors were identified from comparative studies. Meta-analysis was conducted according to PRISMA guidelines using RevMan 5.3. Data are (mean ± SD) prevalence.

**Results:** Systematic review—of 885 abstracts screened, 48 studies (27,760 neonates) were included. The incidence of SSI was 5.6% (1,564 patients). SSI was more frequent in males (61.8%), premature babies (77.4%), and following gastrointestinal surgery (95.4%). Meta-analysis—10 comparative studies (16,442 neonates; 946 SSI 5.7%) showed that predictive factors for SSI development were gestational age, birth weight, age at surgery, length of surgical procedure, number of procedure per patient, length of preoperative hospital stay, and preoperative sepsis. Conversely, preoperative antibiotic use was not significantly associated with development of SSI.

**Conclusions:** Younger neonates and those undergoing abdominal procedures are at higher risk for SSI. Given the lack of evidence-based literature, prospective studies may help determine the risk factors for SSI in neonates.

## Introduction

Surgical site infections (SSI) are infections that occur postoperatively in the area of the body where the surgery took place. SSI can be superficial and involve the skin only, or more serious and involve other tissues, organs, or implanted material. SSI are among the most common hospital acquired diseases and are an important cause of morbidity and mortality in all patients, including neonates and infants (1, 2).

Whilst the incidence and risk factors for SSI in adults and more recently in children have been defined and management guidelines have been established (3, 4), yet little is known about SSI in neonates and infants.

The incidence of SSI is 2–5% in adult patients undergoing inpatient surgery (3). Risk factors associated with SSI included co-morbidities, advanced age, risk indices, patient frailty, and surgery complexity (5).

In children the rate of SSI ranged from 2.5 to 5.4% and dirty wounds, inpatient status, increased duration of surgery, or certain surgical disciplines (cardiovascular, general surgery, neurosurgery, and orthopedics) were associated with increased risk of developing an SSI (4).

Previous studies have shown that the incidence of SSI in neonates and infants can be as high as 17% (1, 2). In this population of patients, several conditions have been reported to be associated with an increased risk of SSI, including admission to the neonatal intensive care unit (NICU), history of prematurity, low birth weight, mechanical ventilation, central venous access, associated co-morbidities, prolonged antibiotic administration, postsurgical hyperglycemia, and neutropenia (1, 2, 6, 7).

In the present study, we aimed to establish the incidence of SSI in neonates and to identifyprognostic factors that may help stratify which neonates are at increased risk to develop this complication. A better understanding of the causes leading to SSI could reduce their incidence, help define guidelines, and eventually improve outcome.

## Methods

To investigate the incidence and risk factors of SSI in neonates, we conducted a systematic review of the literature and complemented it with a meta-analysis of comparative studies. Both the systematic review and the meta-analysis were drafted according to the Preferred Reporting Items for Systematic Reviews and Meta-Analyses (PRISMA) statement (8). The protocol for this systematic review was registered on PROSPERO—international prospective register of systematic reviews (registration number: CRD42017069003) (9). Using a defined search strategy, three investigators (VDC, AB, and GL) independently searched scientific databases (PubMed, Medline, Cochrane Collaboration, Embase, Web of Science, Ovid) using a combination of keywords (Table 1). MeSH headings and terms used were “neonate OR newborn” AND “surgery OR surgical” AND “wound infection OR surgical site infection” (Supplementary File 1).

**Table 1:** Defined search strategy.

**Publication**	
Sources	PubMed, Medline, Cochrane Collaboration, Embase, Web of Science, Ovid
Language	Any
Date	Since 2000
Subject	Human studies
Study type	Retrospective
	Prospective
	Case control
	Cohort
Excluded	Grey Literature
	Case reports
	Case series <10 patients
	Letters
	Editorials
Keywords	Neonate, Neonatal, Newborn
	Surgery
	Surgical site infection
	Surgical wound infection

All gray literature publications (i.e., reports, theses, conference proceedings, bibliographies, commercial documentations, and official documents not published commercially) were excluded. Only studies on neonates (<44 wks gestational age) and/or neonates admitted to the NICU following cervical/thoracic/abdominal surgery published since 2,000 were included. Case reports, case series with <10 patients, animal studies, and opinion articles were excluded. The full text of the potentially eligible studies was retrieved and independently assessed for eligibility by the same three investigators. Any disagreement over the eligibility of particular studies was resolved through discussion with the other two authors (FM and AZ). Outcome measures included demographic data, type and district of surgery, SSI development, preoperative systemic infection, preoperative antibiotic prophylaxis, length of procedure, and number of procedure per patient. Risk factors of SSI were identified from comparative studies.

### Statistical Analysis

Data were analyzed using GraphPad Prism 6.2 Macintosh Version (10). Data were compared using Fisher's exact test and are expressed as mean ± SD. When median and range were reported, mean ± SD were estimated, as previously reported (11).

The meta-analysis was conducted with RevMan 5.3 (12), using the fixed-effects model to produce risk ratio (RR) for categorical variables and mean differences (MD) for continuous variables, along with 95% confidence intervals (CI). We produced *I*^2^ values to assess homogeneity and quantify the dispersion of effect sizes.

### Quality Assessment

Risk of bias for individual studies was assessed in duplicate (VDC and GL) using the methodological index for non-randomized studies (MINORS) (13). Differences between the two reviewers were resolved through consensus and discussion with another author (AZ). The total score for this 12-item instrument ranges 0–24 points with a validated “gold standard” cut-off of 19.8.

Two authors (FM and AZ) independently evaluated the present systematic reviews and meta-analysis using A Measurement Toll to Assess Systematic Reviews (AMSTAR) (14). The PRISMA checklist of our study was then completed (8).

## Results

### Systematic Review

**Figure 1:**
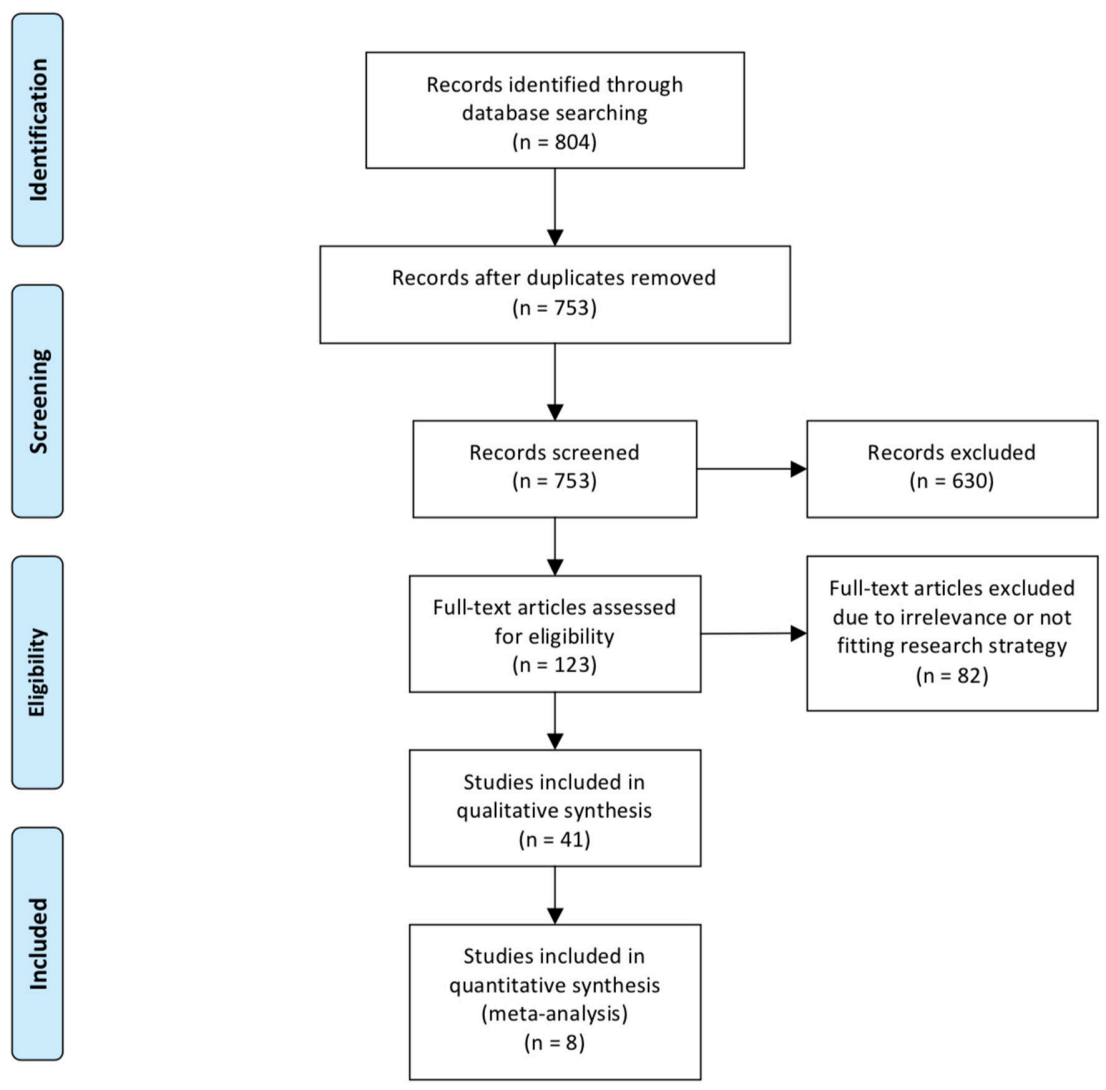
Diagram of workflow in the systematic review and meta-analysis.

Of the 885 abstracts analyzed, 48 full articles, for a total of 27,760 patients (16,517 males, 59.5%) met our inclusion criteria (2, 15–61) (Figure 1). The overall incidence of SSI was 5.6% (*n* = 1,564) with a slight prevalence of male gender (61.8%) and premature babies (77.4%, gestational age at birth: 33 ± 7 weeks). The majority of neonates with SSI had gastrointestinal and/or colorectal surgery (95.4%), followed by thoracic (3.0%), and other (1.6%) procedures.

### Comparative Studies

We analyzed 10 comparative studies (Tables 2a,b) (16–18, 22, 24, 34, 45, 47, 51, 60). Among these articles, there were only two prospective cohort studies (45, 60) and one national prospective database (16). No randomized studies were included. The papers included reported 946 cases of SSI out of 16,442 patients (5.7%). The distribution of surgical districts is significantly different between patients who developed SSI and those who did not (Table 3). SSI development was significantly associated with abdominal surgery (288/2,059 cases, 13.9%) in comparison with other surgical districts. In 13,845 patients the surgical district was not specified. When reported, the most common type of abdominal surgery was laparotomy for congenital abdominal wall defect (601 cases), necrotizing enterocolitis (133), malrotation (33), small bowel atresia (34), volvulus (17), or not specified congenital bowel obstruction (418).

**Table 2a:** Studies comparing SSI *versus* no-SSI neonates (preoperative data).

**References**	**Year**	**Type of study**	**SSI (No.) [%]**	**Gestational age(weeks)**		**Birth weight (grams)**		**Preoperative hospitalstay (days)**		**Preoperative sepsis(No.) [%]**		**Preoperativeantibioticsprophylaxis (No.) [%]**	
				**SSI**	**No-SSI**	**SSI**	**No-SSI**	**SSI**	**No-SSI**	**SSI**	**No-SSI**	**SSI**	**No-SSI**
Garcia et al. (24)	2005	R	125/279 [44.8]	38 (26–41)^§^	36 (24–40)^§^	2,700 (625–4,200)^§^	2,108 (700–4,200)^§^	2 (1–119)^§^	2 (1–105)^§^	77 [61.6]	72 [46.7]	n.r.	n.r.
Baird et al. (16)	2012	PD	48/395 [12.1]	36 (r 30–40)	36 (r 25–41)	n.r.	n.r.	n.r.	n.r.	n.r.	n.r.	n.r.	n.r.
Rojo R (47)	2012	R	40/90 [44.4]	32 (25–40)^§^	34 (24–40)^§^	2,121 ± 945	2,546 ± 1,179	3 (0–90)^§^	0.25 (0–120)^§^	20 [50]	20 [40]	36 [90]	40 [80]
Lejus et al. (34)	2013	R	11/286 [3.8]	37 (35–38)^#^	38 (37–39)^#^	2,530 (2,340–3,115)^#^	3,000 (2,719–3,500)^#^	n.r.	n.r.	3 [27.3]	18 [6.6]	7 [63.6]	94 [34.2]
Segal et al. (51)	2014	R	31/498 [6.2]	33 ± 6	33 ± 6	2,190 ± 1,127	2,152 ± 1,158	n.r.	n.r.	n.r.	n.r.	n.r.	n.r.
Battin M (18)	2016	R	10/60 [16.7]	30 (24–36)^#^	34 (26–37)^#^	1,002 (750–2,633)^#^	2,170 (970–2,875)^#^	n.r.	n.r.	n.r.	n.r.	7 [70]	41 [84]
Clements et al. (22)	2016	R	58/188 [30.8]	33 (r 24–40)	33 (r 23–42)	2,042 ± 1,165	1,993 ± 1,156	43.8	27.2	n.r.	n.r.	27 [47]	68 [52]
Prasad et al. (45)	2016	P	38/738 [5.1]	37 (34–38)^#^	38 (36–39)^#^	2,537 (1,955–3,265)^#^	2,945 (2,365–3,400)^#^	n.r.	n.r.	n.r.	n.r.	n.r.	n.r.
Bartz-Kurycki et al. (17)	2018	R	542/13,589 [3.9]	n.r.	n.r.	n.r.	n.r.	n.r.	n.r.	27 [4.9]	274 [2.1]	n.r.	n.r.
Woldemicael et al. (60)	2018	P	43/319 [13.5]	n.r.	n.r.	2,121 ± 887	2,083 ± 1,082	7 (4–17)^#^	3 (1–15)^#^	n.r.	n.r.	n.r.	n.r.

R, retrospective; PD, prospective database; P, prospective cohort study; n.r, not reported;

§ , median (range);

# *, median (IQR); r, range*.

**Table 2b:** Studies comparing SSI *vs*. no-SSI neonates (intra and postoperative data).

**References**	**Age at surgery(days)**		**Operative time(minutes)**		**Number ofprocedures**		**Length of hospitalstay (days)**	
	**SSI**	**No-SSI**	**SSI**	**No-SSI**	**SSI**	**No-SSI**	**SSI**	**No-SSI**
Garcia et al. (24)	n.r.	n.r.	75 (20–240)^§^	50 (10–300)^§^	n.r.	n.r.	n.r.	n.r.
Baird et al. (16)	n.r.	n.r.	n.r.	n.r.	n.r.	n.r.	95.9 (r 21–349)	41.6 (r 1–194)
Rojo et al. (47)	21 (0–120)^§^	12.5 (0–150)^§^	100.8 ± 49.6	108 ± 51.6	n.r.	n.r.	n.r.	n.r.
Lejus et al. (34)	7 (2–14)^#^	11 (0–20)^#^	70 (64–123)^#^	44 (22–79)^#^	n.r.	n.r.	n.r.	n.r.
Segal et al. (51)	85 (5–120)^#^	11 (4–52)^#^	n.r.	n.r.	2 (1–4)^#^	1 (1–2)^#^	79 (34–131)^#^	25 (9–70)^#^
Battin et al. (18)	10 (2–81)^#^	5 (2–12)^#^	110 (60–134)^#^	68 (53–96)^#^	n.r.	n.r.	n.r.	n.r.
Clements et al. (22)	n.r.	n.r.	108 ± 62	86 ± 58	2.44 ± 1.33 (r 1–6)	1.42 ± 0.73 (r 1–5)	n.r.	n.r.
Prasad et al. (45)	5.5 (2–14.5)^#^	5 (2–11)^#^	n.r.	n.r.	n.r.	n.r.	n.r.	n.r.
Bartz-Kurycki et al. (17)	n.r.	n.r.	78 (46–132)^#^	64 (34–112)^#^	n.r.	n.r.	n.r.	n.r.
Woldemicael et al. (60)	8 (5–28)^#^	6 (2–45)^#^	n.r.	n.r.	n.r.	n.r.	40 (28–124)^#^	26.5 (14–76)^#^

n.r., not reported;

§ , median (range);

# *median (IQR); r, range*.

**Table 3:** Incidence of SSI according to the surgical district.

**Surgical districts**	**SSI (n)**	**No-SSI (*n*)**	***p***
Neck	6	78	*p* < 0.01
Thorax	21	280	
Abdomen	288	1,771	
Pelvis	4	115	
Perineum	3	31	
Not specified	624	13,221	
Total	946	15,496	

### Meta-Analysis

**Figure 2:**
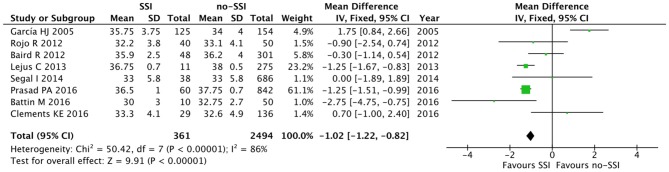
Forest plot comparison of gestational age at birth of neonates with or without postoperative SSI.

**Figure 3:**
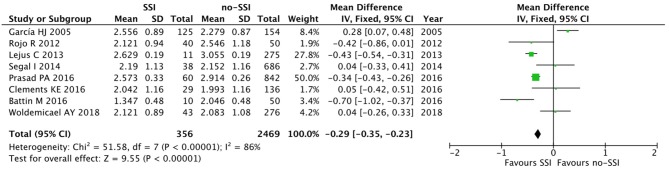
Forest plot comparison of birth weight of neonates with or without postoperative SSI.

**Figure 4:**
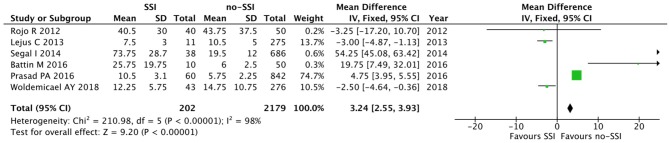
Forest plot comparison of age at procedure of neonates with or without postoperative SSI.

**Figure 5:**
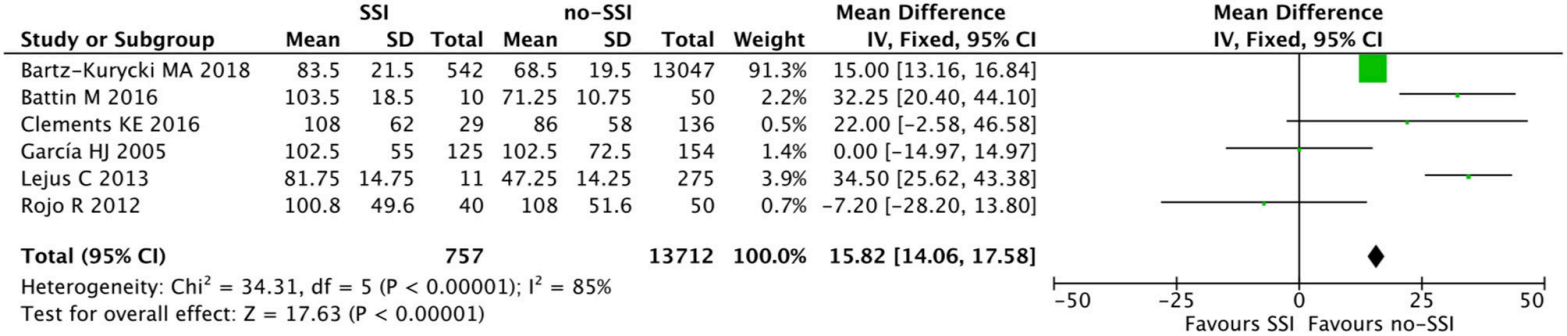
Forest plot comparison of length of procedure of neonates with or without postoperative SSI.

**Figure 6:**
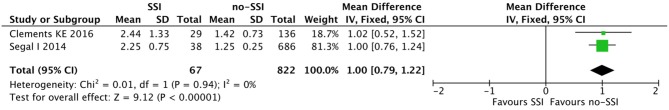
Forest plot comparison of number of procedure per patient in neonates with or without postoperative SSI.

**Figure 7:**
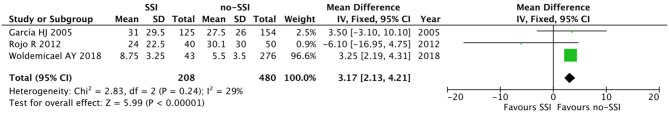
Forest plot comparison of preoperative hospital stay in neonates with or without postoperative SSI.

**Figure 8:**
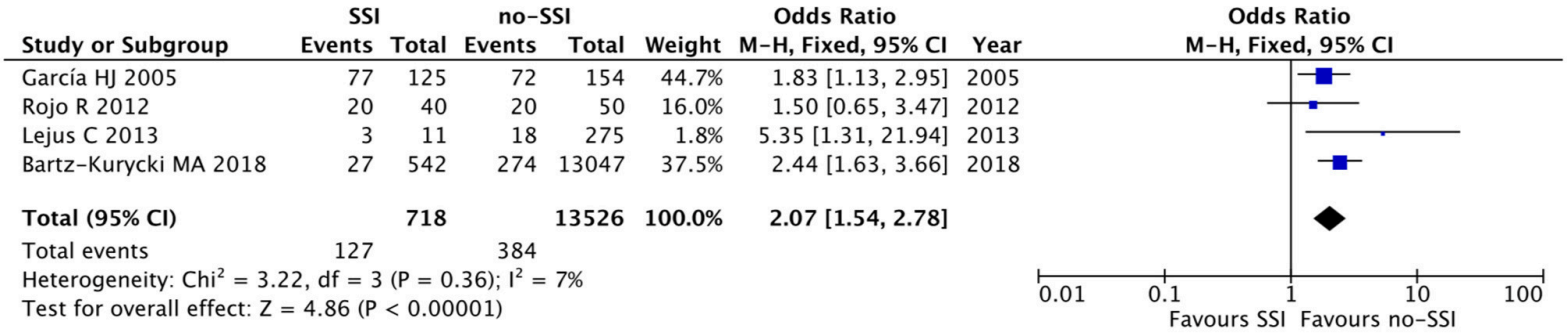
Forest plot comparison of preoperative systemic infection in neonates with or without postoperative SSI.

**Figure 9:**
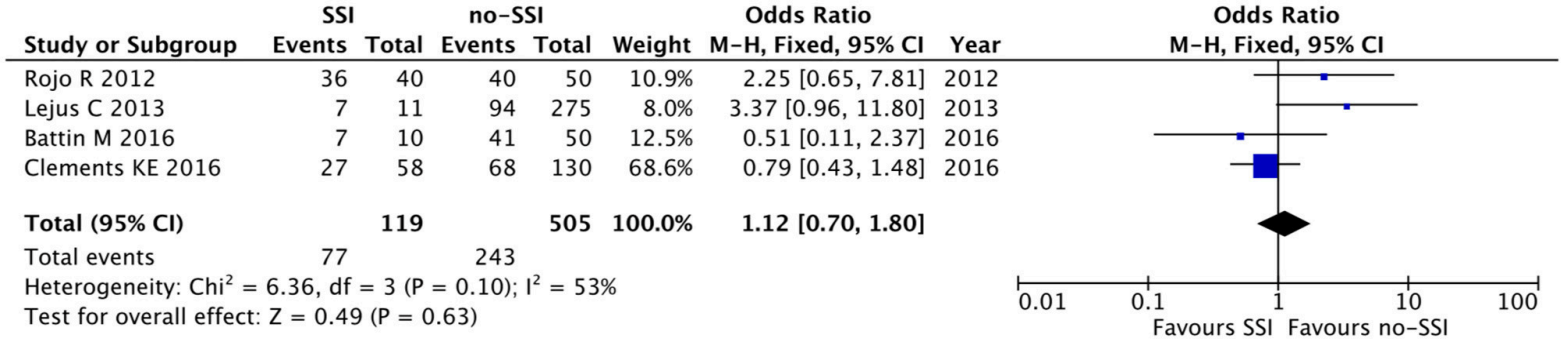
Forest plot comparison of preoperative antibiotic prophylaxis in neonates with or without postoperative SSI.

**Figure 10:**
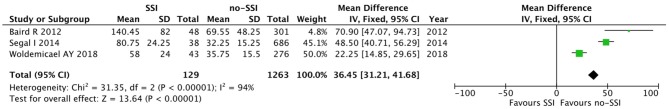
Forest plot comparison of length of hospital stay in neonates with or without postoperative SSI.

The meta-analysis of the 10 comparative studies (16–18, 22, 24, 34, 45, 47, 51, 60) showed that there was a slight albeit significant difference between neonates with and without SSI for gestational age (34.2 ± 2.4 weeks vs. 34.7 ± 2.3; *p* < 0.00001, MD −1.02, 95% CI [−1.22, −0.82], *I*^2^ = 86%; Figure 2) and birth weight (2,171 ± 479 grams vs. 2,384 ± 411; *p* < 0.00001, MD −0.29, 95% CI [−0.35, −0.23], *I*^2^ = 86%; Figure 3). Neonates with SSI were older at surgery compared to those without SSI (28.4 ± 24.4 days vs. 16.7 ± 14.3; *p* < 0.00001, MD 3.24, 95% CI [2.55, 3.93], *I*^2^ = 98%; Figure 4). The other main predictive factors for the development of an SSI were length of surgical procedure (SSI 96.7 ± 11.2 min vs. no SSI 71.2 ± 20.8; *p* < 0.00001, MD 15.82, 95% CI [14.06, 17.58], *I*^2^ = 85%; Figure 5), number of procedure per patient (SSI 2.3 ± 0.1 procedures vs. no SSI 1.3 ± 0.1; *p* < 0.00001, MD 1.00, 95% CI [0.79, 1.22], *I*^2^ = 0%; Figure 6), length of preoperative hospital stay (SSI 21.3 ± 11.4 days vs. no SSI 21.0 ± 13.5; *p* < 0.00001, MD 3.17, 95% CI [2.13, 4.21], *I*^2^ = 29%; Figure 7), and preoperative systemic infection (SSI 127/718 neonates, 17.7% vs. no SSI 384/13,526, 2.8%; *p* < 0.00001, OR 2.07, 95% CI [1.54, 2.78], *I*^2^ = 7%; Figure 8). Conversely preoperative antibiotics prophylaxes were not significantly associated with a reduced development of SSI (SSI 77/119 neonates, 64.7% vs. no SSI 243/505, 48.1%; *p* = 0.63, OR 1.12, 95% CI [0.70, 1.80], *I*^2^ = 53%; Figure 9). As expected, neonates with SSI showed a significant lengthened hospital stay in comparison with those without SSI (SSI 93.1 ± 42.6 days vs. no SSI 45.8 ± 20.6; *p* < 0.00001, MD 36.45, 95% CI [31.21, 41.68], *I*^2^ = 94%; Figure 10).

## Discussion

The overall rate of SSI in adult and pediatric patients is ~ 2–5% and SSI are associated with increased morbidity, mortality, healthcare costs, and length of hospital stay (3, 62). Risk factors for SSI have been identified in predominantly adult populations and include advanced age, hyperglycemia, malnutrition, co-morbidities, risk indices, patient frailty, prior infections, complexity of surgery, increased operative time, and increased blood loss during surgery (5, 45). With regard to children, certain surgical disciplines (cardiovascular, general surgery, neurosurgery, and orthopedics) were associated with increased risk of developing an SSI (4). SSI development substantially increases the clinical and economic burden of surgery, because of prolonged hospitalization of the patient, diagnostic tests, and treatment. Moreover, SSI negatively impact on patient physical and mental health as well as on loss of earnings during recovery (63, 64).

Our study shows that neonates undergoing abdominal surgery are at high risk of SSI and that age at surgery and length of procedure are the main predictors of SSI in those admitted to NICU. Similarly, male gender and gestational age may be associated to the development of SSI, but the present data are not conclusive.

Although there is an abundance of literature on SSI in adults, there is a lack of studies having examined risk factors for SSI in neonates undergoing surgical interventions.

In the present systematic review of the literature on more than 27,000 patients, we found an incidence of SSI of 5.5%. Interestingly, the overall rate of SSI in the neonatal age group in our review is comparable to the rates reported in the older pediatric age group (51, 65, 66). This finding suggests that neonates may be less prone to SSI than it might be expected based on their alleged fragility, as they represent a special population that is thought to be at higher risk for infection due to their immature immune systems (6, 67, 68).

According to our study, premature infants represent a significant proportion of infants who require surgical interventions (51), and this is confirmed by the overall prevalence of SSI in this cohort (77%). When examining the type of surgical intervention, the vast majority of infants underwent gastrointestinal surgery (96%). In particular, abdominal surgery was significantly associated with an increased risk of SSI (23, 54). The most common type of surgical intervention described in the included articles was laparotomy for congenital abdominal wall defects, necrotizing enterocolitis, or congenital bowel obstruction. This surgical procedure is known to compromise the integrity of the gastrointestinal tract and to potentially result in bacterial translocation. Surgical wounds following a neonatal laparotomy are classified, at best, as clean-contaminated wounds, which justify the highest prevalence of SSI in this sub-group (22). The Canadian Pediatric Surgery Network recently reported an overall 15% incidence of SSI in infants with gastroschisis who underwent immediate (<6 h after birth) or delayed closure (55). For this reason, to reduce time of visceral exposure, the authors have proposed the gastroschisis sutureless closure, as it is also associated with a reduced risk of SSI (54).

To define the risk factors that are to be considered by the surgeon to estimate the risk of SSI at the time of neonatal surgery, we selected comparative studies that analyzed neonates with or without SSI. Interestingly, we observed that neonates who developed SSI had a younger GA compared to those who did not. This was a validation of the results from the systematic review that showed a high prevalence of prematurity among patients who developed SSI. Likewise, also a lower birth weight was associated with an increased risk of SSI in the included studies, even if the SSI group had an older age at the time of their procedures compared to the group who neonates who did not develop SSI. Moreover, this group also had a longer preoperative length of stay and it likely required a greater number of invasive diagnostic and therapeutic procedures per patient, as already reported by Garcia and Lejus (24, 34). In fact, this study confirmed a significant difference in number of surgical procedures between neonates with SSI and those without. This figure may be related to the severity of illness, with sicker patients being more likely to require additional procedures, although one third of SSI did occur after a single or first procedure. A long preoperative admission is a risk factor for SSI, as it is proportional to the severity of the underlying clinical conditions, the need for invasive devices and treatments (included prolonged antibiotics), and it promotes nosocomial flora colonization. Furthermore, neonates admitted to NICU are highly susceptible to nosocomial infections. As an expected consequence, the presence of a systemic infection significantly influenced the SSI incidence in our study.

Our present study also highlighted that the length of surgery is another risk factor for SSI. Length of surgery was not identified by all studies as a risk factor for SSI, and our meta-analysis confirmed the findings of Clements and coworkers who found a longer operative time in patients developing SSI (22). Prolonged visceral exposure may negatively impact on surgical outcome as consequence of skin contamination. Furthermore, the longer the operative time the deeper the surgical stress response, as the invasiveness of surgery and the length of procedure significantly correlated to oxidative stress activation and cortisol response (69–71). The latter may have an impact on postoperative outcome, such as the development of infectious complications, including SSI.

This study did not show any difference in preoperative antibiotic administration between neonates who developed SSI and those who did not. Whilst standardized preoperative antibiotic protocols in the adult population have shown to reduce the rate of SSI, a consensus is lacking among pediatric surgeons regarding preoperative antibiotic prophylaxis especially in neonates (1, 4). The different definitions of antibiotic prophylaxis and antibiotic regimen used in the analyzed studies may have led to our findings. The isolation of skin flora from a large number of wound cultures suggests that standardization of preoperative prophylaxis could potentially have an impact on the rate of SSI as has previously been demonstrated in pediatric patients (72, 73).

## Limitations of the Study

Our study has some limitations. The first relates to the relative small number of studies available for the meta-analysis, with only two prospective cohort study and one national prospective database included. Nonetheless, the population of neonates reported in the studies was not small, with more than 27,000 neonates included.

The second limitation is that we could not analyze important variables, such as the use of adequate antibiotic prophylaxis, as they were not reported in the few studies selected in our analysis. Finally, the third limitation is the relative heterogeneity of the patient population: although we tried to limit the study to neonates, we included both patients with a post-conceptional age below 44 weeks and infants admitted in the NICU following abdominal, cervical, and thoracic surgery.

As a consequence, in our meta-analysis, none of the studies reached the gold standard cut-off on MINORS of 19.8 out of 24 (Table 4). However, when independently assessed by two authors using AMSTAR, the present systematic reviews and meta-analysis received a relevant score (Supplementary File 2) and the PRISMA checklist was completed (Supplementary File 3).

**Table 4:** Risk of bias assessment for individual studies using methodological index for nonrandomized studies (MINORS) (13).

**Item**	**Garcia et al. (24)**	**Bair et al. (16)**	**Rojo et al. (47)**	**Lejus et al. (34)**	**Segal et al. (51)**	**Battin (18)**	**Clements et al. (22)**	**Prasad et al. (45)**	**Bartz-Kurycki et al. (17)**	**Woldemicael et al. (60)**
1. A clearly stated aim	2	2	2	2	2	2	2	2	2	2
2. Inclusion of consecutive patients	2	2	2	2	2	2	2	2	2	2
3. Prospective collection of data	0	2	0	0	0	0	0	2	0	2
4. Endpoints appropriate to the aim of the study	2	2	2	2	2	2	2	2	2	2
5. Unbiased assessment of the study endpoint	0	0	0	0	0	0	0	0	0	0
6. Follow-up period appropriate to the aim of the study	0	0	0	0	0	0	0	0	0	0
7. Loss to follow-up less than 5%	0	0	0	0	0	0	0	0	0	0
8. Prospective calculation of the study size	0	0	0	0	0	0	0	0	0	0
9. An adequate control group	2	2	2	2	2	2	2	2	2	2
10. Contemporary groups	2	2	2	2	2	2	2	2	2	2
11. Baseline equivalence of groups	1	2	1	2	2	2	2	2	2	2
12. Adequate statistical analyses	2	2	2	2	2	2	2	2	2	2
Total score	13	16	13	14	14	14	14	16	14	16

## Conclusion

In conclusion, SSI is a significant complication in neonates admitted to NICU that can negatively impact their outcome by prolonging hospital stay and increasing the risk for further complications, such as potentially fatal sepsis. Younger and smaller neonates at birth, those requiring longer or multiple operative procedures, and those with prolonged preoperative hospital stay and preoperative sepsis are at higher risk for SSI. These patients require special attention with close monitoring during their post-operative course. Given the lack of evidence in the literature, well-designed prospective studies on large cohorts of neonates may help setting up specific guidelines for the prevention and treatment of SSI in this particular population.

## Data Availability

All datasets generated for this study are included in the manuscript and/or the Supplementary Files.

## Author Contributions

VC, AB, GL, FM, and AZ: conception and design, analysis and interpretation, drafting, final approval. VC, AB, and GL: data acquisition. VC, GL, FM, and AZ: quality assessment. GL, FM, and AZ: revision.

### Conflict of Interest Statement

The authors declare that the research was conducted in the absence of any commercial or financial relationships that could be construed as a potential conflict of interest.

## Abbreviations

SSI: surgical site infection
NICU: neonatal intensive care unit.
