# Supplementary material for: Risk Factors for Surgical Site Infection in Neonates: A Systematic Review of the Literature and Meta-Analysis
Source: Front Pediatr. 2019 Mar 29;7:101. doi: 10.3389/fped.2019.00101 (PMC6449628; doi:10.3389/fped.2019.00101)
Supplement: Supplementary file 1 [file Data_Sheet_1.doc]

**Supplementary file 1:** Search strategy.

**PubMed/Medline**

1. (neonate*adj2 OR neonatal*adj2 OR newborn*adj2).mp.
2. surgery OR surgical
3. (wound infect*adj2 OR surgical site infect*adj2).mp.
4. 1 AND 2 AND 3

**Cochrane**

1. (neonate*adj2 OR neonatal*adj2 OR newborn*adj2).mp.
2. surgery OR surgical
3. (wound infect*adj2 OR surgical site infect*adj2).mp.
4. 1 AND 2 AND 3

**EMBASE**

1. exp (neonate*adj2 OR neonatal*adj2 OR newborn*adj2).mp.
2. surgery OR surgical
3. exp (wound infect*adj2 OR surgical site infect*adj2).mp.
4. 1 AND 2 AND 3
