# Supplementary material for: Risk Factors for Surgical Site Infection in Neonates: A Systematic Review of the Literature and Meta-Analysis
Source: Front Pediatr. 2019 Mar 29;7:101. doi: 10.3389/fped.2019.00101 (PMC6449628; doi:10.3389/fped.2019.00101)
Supplement: Supplementary file 2 [file Data_Sheet_2.doc]

**Supplementary file 2: AMSTAR criteria for the present systematic reviews and meta-analysis assessed by two senior authors.**

| Item | FM | AZ |
| --- | --- | --- |
| 1. Was an 'a priori' design provided? | 1 | 1 |
| 2. Was there duplicate study selection and data extraction? | 1 | 1 |
| 3. Was a comprehensive literature search performed? | 1 | 1 |
| 4. Was the status of publication (i.e. grey literature) used as an inclusion criterion? | 1 | 1 |
| 5. Was a list of studies (included and excluded) provided? | 0 | 0 |
| 6. Were the characteristics of the included studies provided? | 1 | 1 |
| 7. Was the scientific quality of the included studies assessed and documented? | 1 | 1 |
| 8. Was the quality of the included studies used appropriately in formulating conclusions? | 1 | 0 |
| 9. Were the methods used to combine the findings of studies appropriate? | 1 | 1 |
| 10. Was the likelihood of publication bias assessed? | 1 | 1 |
| 11. Was the conflict of interest included? | 0 | 0 |
| Total | 9/11 | 8/11 |

0 = No, 1 = Yes
